# Maternal Obesity during Gestation Impairs Fatty Acid Oxidation and Mitochondrial SIRT3 Expression in Rat Offspring at Weaning

**DOI:** 10.1371/journal.pone.0024068

**Published:** 2011-08-25

**Authors:** Sarah J. Borengasser, Franchesca Lau, Ping Kang, Michael L. Blackburn, Martin J. J. Ronis, Thomas M. Badger, Kartik Shankar

**Affiliations:** 1 Department of Pediatrics, University of Arkansas for Medical Sciences, Little Rock, Arkansas, United States of America; 2 Department of Pharmacology and Toxicology, University of Arkansas for Medical Sciences, Little Rock, Arkansas, United States of America; 3 Department of Physiology and Biophysics, University of Arkansas for Medical Sciences, Little Rock, Arkansas, United States of America; 4 Arkansas Children's Nutrition Center, Little Rock, Arkansas, United States of America; Universita Magna-Graecia di Catanzaro, Italy

## Abstract

*In utero* exposure to maternal obesity increases the offspring's risk of obesity in later life. We have also previously reported that offspring of obese rat dams develop hepatic steatosis, mild hyperinsulinemia, and a lipogenic gene signature in the liver at postnatal day (PND)21. In the current study, we examined systemic and hepatic adaptations in male Sprague-Dawley offspring from lean and obese dams at PND21. Indirect calorimetry revealed decreases in energy expenditure (p<0.001) and increases in RER values (p<0.001), which were further exacerbated by high fat diet (45% kcals from fat) consumption indicating an impaired ability to utilize fatty acids in offspring of obese dams as analyzed by PRCF. Mitochondrial function is known to be associated with fatty acid oxidation (FAO) in the liver. Several markers of hepatic mitochondrial function were reduced in offspring of obese dams. These included SIRT3 mRNA (p = 0.012) and mitochondrial protein content (p = 0.002), electron transport chain complexes (II, III, and ATPase), and fasting PGC-1α mRNA expression (p<0.001). Moreover, hepatic LCAD, a SIRT3 target, was not only reduced 2-fold (p<0.001) but was also hyperacetylated in offspring of obese dams (p<0.005) suggesting decreased hepatic FAO. In conclusion, exposure to maternal obesity contributes to early perturbations in whole body and liver energy metabolism. Mitochondrial dysfunction may be an underlying event that reduces hepatic fatty acid oxidation and precedes the development of detrimental obesity associated co-morbidities such as insulin resistance and NAFLD.

## Introduction

The obesity epidemic continues to worsen worldwide, with the most alarming increases occurring in children [1]. If the current trends of childhood obesity continue, it is projected that 60 million children will be overweight or obese by 2020 worldwide [1]. Obesity in children is not only becoming more prevalent, but is also beginning at younger ages, even as young as infants (0–11 mo) [2], [3]. Accelerated growth during infancy and perhaps even *in utero* programs not only increased susceptibility for obesity in later life, but also increases the risk of several obesity-related co-morbidities, such as insulin resistance and cardiovascular disease [4]–[6]. This occurrence of early onset obesity suggests that the intrauterine environment may be contributing to the obesity epidemic through fetal programming of offspring metabolism and disruption of energy balance [7], [8].

Using a rat model of gestational obesity, we have previously shown that maternal obesity, at the time of conception, leads to greater fat mass, increased body fat percentage, and insulin resistance in the offspring in later life (postnatal day (PND) 130), and worsens when challenged with a high fat diet (HFD) [9]. Further, indications of metabolic abnormalities in these offspring are apparent as early as PND21 and include hepatic steatosis, mild hyperinsulinemia, and a lipogenic gene signature in the liver [10]. It is possible that maternal obesity-induced exposure to elevated fatty acids *in utero* leads to a shunting of fatty acids towards lipogenesis and away from fatty acid oxidation. However, the precise mechanisms that contribute to increased susceptibility of offspring from obese dams to develop nonalcoholic fatty liver disease (NAFLD) in early life, and obesity in later life, remain poorly understood.

Hepatic mitochondria are of maternal origin, and as such, may be an important target to consider for investigating metabolic perturbations in offspring of obese women. Mitochondria are critical sites of metabolism and are associated with energy sensing. For example, mitochondrial dysfunction in the liver has been associated with the development of NAFLD in obese rats, as shown by: reduced fatty acid oxidation; decreased cytochrome c protein content in the liver [11], [12]; and decreased carnitine palmitoyl-CoA transferase-1 activity [11]. Moreover, maternal exposure to a high fat diet prior to conception, and during gestation and lactation, has been reported to lead to the development of NAFLD and insulin resistance [13] in adult offspring that was linked to reduced mitochondrial electron transport chain activity in mice [14]. Furthermore, mitochondrial dysfunction has been linked to human patients diagnosed with NAFLD [15].

In the current study, we examined systemic and hepatic metabolic adaptations in offspring from lean and obese dams at PND21. First, we studied whether maternal obesity alters energy expenditure and substrate utilization in offspring using indirect calorimetry. Second, we sought to determine the role of mitochondrial function in offspring by measuring gene expression and protein content of key mitochondrial markers in the liver. Third, we investigated fasting-induced changes in hepatic mitochondrial markers involved in energy status. Our results demonstrate that offspring from obese rat dams have increased susceptibility to develop systemic and hepatic energy utilization perturbations that are mediated, in part, by mitochondrial dysfunction at weaning.

## Materials and Methods

### Animals and chemicals

Female Sprague-Dawley rats (150–175g) were obtained from Charles River Laboratories (Wilmington, MA). Animals were housed in an AAALAC-approved animal facility in a temperature and light controlled room (12 h light-12 h dark cycle). All experimental protocols were approved by the Institutional Animal Care and Use Committee at the University of Arkansas for Medical Sciences (Protocol # 2971). Unless specified, all chemicals were obtained from Sigma-Aldrich Chemical Co. (St. Louis, MO).

### Experimental protocol

Virgin female Sprague-Dawley rats were intragastrically cannulated and allowed to recover for 10 d as previously described [9], [16]–[19]. Rats were fed liquid diets at either 155 kcal/kg^3/4^ · day (referred to as **lean dams**) or at 220 kcal/kg^3/4^ · d (40% excess calories, referred to as **obese dams**). We have previously reported body weights and body compositions of lean and obese dams [9]. Total enteral nutrition (TEN) diets met National Research Council (NRC) nutrient recommendation and have been used previously by our group [9], [16]–[22]. Infusion of diets was carried out for 23 h/d using computer controlled pumps for 3 wk. Animals had *ad libitum* access to drinking water and body weights were measured three times per week. Following 3 wk of overfeeding to induce obesity in the 220 kcal/kg^3/4^ · d group, lean and obese rats (N = 15/group) were allowed to mate for 1 wk. Each female rat was housed with one lean breeder male and allowed *ad libitum* access to AIN-93G diet during this period. After mating all female rats (lean and obese) received diets at 220 kcal/kg^3/4^ · d (NRC recommended caloric intake for pregnancy in rats). All rats were allowed to give birth naturally. Numbers and sex of pups, birth weight, and crown-to-rump and anogenital distance were measured for each pup on PND1 as previously described [9], [10]. On PND2, four male and four female pups from each litter were cross-fostered to lean dams that had been previously time-impregnated to give birth on the same day as the obese dams receiving infusion diets. Cross-fostered dams were not cannulated and had *ad libitum* access to AIN-93G pelleted diets throughout lactation. Using this experimental paradigm, we ensured that offspring's exposure to any effects of maternal obesity was limited almost exclusively to the intrauterine environment [9]. Female offspring of lean and obese dams were used for separate experiments, and only data from male offspring are reported here. Male offspring were euthanized under anesthesia at PND21 (N = 15/group). In some experiments, animals were sacrificed following a 24 h (9:00 am – 9:00 am) fast. At sacrifice, liver was weighed, formalin fixed, and immediately frozen in liquid nitrogen and stored at −70°C for later analyses. Serum was obtained by centrifugation of blood samples and stored at −20°C.

### Indirect calorimetry

Offspring from lean and obese rat dams (N = 5/group from separate litters) at PND21 were housed under conditions of 12∶12 h light-dark cycle in metabolic chambers using the Complete Lab Animal Monitoring system (CLAMS) to assess energy expenditure (EE), respiratory exchange ratio (RER), physical activity, and food intake (Columbus Instruments, Columbus, OH). Offspring were housed in the CLAMS chambers from PND20 – PND51. Rats were acclimated to the metabolic chambers for a minimum of 7 days while having *ad libitum* access to the AIN-93G diet. Rats had *ad libitum* access to the AIN-93G or high fat diet (HFD, 45% kcals from fat) throughout the CLAMS measurement period. Data from 3 consecutive 24-h cycles for both AIN-93G (PND34 – PND 37) and high fat diet (PND48 – PND51) were converted into percent relative cumulative frequency (PRCF) values. Expressing indirect calorimetry data as PRCF has been shown to discern small changes in EE and RER values that may be missed by averaging values over 24-h periods [23]. EE was calculated using a modified Weir equation [24], [25]: EE = Calorific value (CV) · VO_2subject_, CV = 3.815 + 1.232 · respiratory exchange ratio (RER). Physical activity was measured as the total number of infrared beam breaks in the X, Y, and Z axes during 20 min intervals. Percent relative cumulative frequency (PRCF) was used to analyze EE and RER values as previously described [23]. Briefly, EC50 values were derived following nonlinear regression using 4-parameter Hill plot.

### RNA isolation and Real-time RT-PCR

Total RNA was isolated from liver of offspring at PND21 (N = 15/group) using RNeasy mini columns (QIAGEN, Valencia, CA) including on-column DNase digestion. One microgram of total RNA was reverse transcribed using IScript cDNA synthesis kit (BioRad, Hercules, CA). Real-time PCR analysis was performed using itaq SYBR Green Supermix (Biorad, Hercules, CA) with each sample run in singlet as described previously using an ABI Prism 7500 instrument [17], [22]. Gene specific primers were designed using Primer Express Software for sirtuins (SIRT) 1, 2, 3, 4, 5, 6, and 7 and peroxisome proliferator-activated receptor gamma-coactivator (PGC)-1α (**Table 1**). Relative amounts of mRNA were quantified using a standard curve run in duplicates and normalized to the expression of SRP14.

**Table 1:** Primers Sequences for Real-time RT-PCR Analyses.

Gene Name	Forward primer (5′- 3′)	Reverse primer (5′- 3′)
**SIRT1**	CTGTTTCCTGTGGGATACCTGACT	ATCGAACATGGCTTGAGGATCT
**SIRT2**	TCCACTGGCCTCTATGCAAACT	GCAAAGAAGGGTTCTGGATGTT
**SIRT3**	GGCACTACAGGCCCAATGTC	TCTCTCAAGCCCGTCGATGT
**SIRT4**	TTACAGCGCTTCATTAGCCTTTC	CCCACCTTTTCTGACCTGTAGTCT
**SIRT5**	AGAGCAAGATCTGCCTCACCAT	AGCCCCCGAGATGATGACTAT
**SIRT6**	CCGTCTGGTCATTGTCAACCT	GCTTCATGAGCTTGCACATCAC
**SIRT7**	GGGTCCAGCTTGAAGGTACTGA	GTCCACTGCAGGTTCACAATGT
**PGC-1α**	CTACAATGAATGCAGCGGTCTT	TGCTCCATGAATTCTCGGTCTT

Gene specific primers were designed using Primer Express™ Software (Applied Biosystems, Foster city, CA). Real-time PCR reactions were carried out according to manufacturer's instructions for 2X SYBR green master mix and monitored on a ABI Prism 7500 sequence detection system (Applied Biosystems, Foster city, CA) as described under methods. SIRT: sirtuin; PGC-1α: peroxisome proliferator activated receptor-γ coactivator-1α.

### Immunoblotting and Immunoprecipitation

Total lysates from liver tissue was prepared in RIPA buffer (25 mM Tris-HCl, 150 mM NaCl, 1.0% NP-40, 1.0% deoxycholic acid, 0.1% SDS, 2 mM EDTA) containing 1 mM PMSF and protease inhibitors (Sigma, St. Louis, MO). Mitochondrial protein extracts were prepared using a Mitochondrial Isolation Kit for Tissue (Pierce, Rockford, IL). Quantification of proteins was performed using BCA assay (Pierce, Rockford, IL). Immunoblotting was performed for oxidative phosphorylation complexes I–V (MitoSciences, Eugene, OR), PGC-1α (Calbiochem, La Jolla, CA), SIRT3 (Cell Signaling Technology, Beverly, MA), long chain acyl-CoA dehydrogenase (LCAD) (gift from Dr. Gerard Vockley, University of Pittsburgh), and voltage-dependent anion channel-1 (VDAC1) (Abcam, Cambridge, MA) as previously described in either total liver lysates or extracts from mitochondrial fractions (N = 4–8/group) [26]. Immunoprecipitation was performed using a commercially available kit (Catch and Release, Millipore, Billerica, MA). Briefly, 100 µg of protein from pooled liver mitochondrial fractions (each pool representing 2 separate animals) were performed (N = 4/group). Following overnight incubation with LCAD or nonspecific IgG (Santa Cruz Biotechnology, Santa Cruz, CA) immune complexes were solubilized in 1 X SDS buffer. Aliquots were resolved using SDS-PAGE and immunoblotting was performed using acetylated-lysine antibody (Cell Signaling Technology, Beverly, MA). Detection of immunoblots was carried out using HRP-linked secondary antibodies (Santa Cruz Biotechnology, Santa Cruz, CA) followed by chemiluminescence (West Pico, Pierce, Rockford, IL). Desitometric quantitation of immunoblots was performed using Quantity One software (BioRad, Hercules, CA).

### Statistical Analysis

Data are expressed as means ± SEM, significance was set at p<0.05. Differences between offspring of lean and obese dams at PND21 were determined using two-tailed Student's *t*-test. Differences between offspring of lean and obese dams at PND21 fed a AIN-93G or HFD were analyzed using two-way Analysis of variance followed by all-pair wise comparisons by Fisher least significant difference (LSD). Statistical analyses were performed using SigmaPlot 11.0 software (Systat Software Inc., San Jose, CA).

## Results

### Exposure to Maternal Obesity Decreases Energy Expenditure (EE) and Respiratory Exchange Ratio (RER) in the Offspring

**Figure 1:**
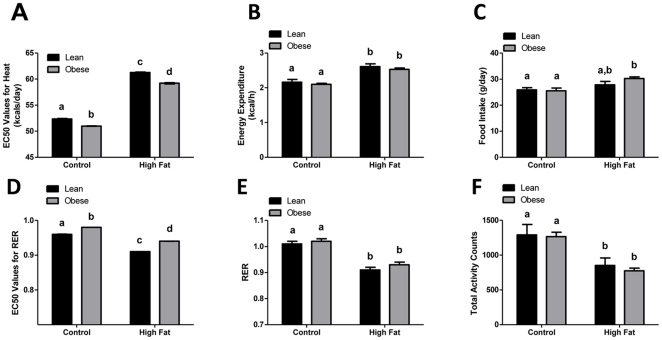
Indirect calorimetry of offspring from lean and obese rat dams. (**A**) EC50 values for energy expenditure (kcal/day) and (**B**) Respiratory exchange ratio (RER) as shown by PRCF analysis in the offspring of lean and obese rat dams (N = 5 per group) fed either an AIN-93G or high fat diet (45% kcals from fat) *ad libitum*. EC50 values were also included as means±SE. Different letter superscripts indicate statistical significance (p<0.05). (**C**) 24-hr averaged values of energy expenditure, (**D**) RER, (**E**) total activity counts, and (**F**) food intake are shown from offspring of lean and obese rat dams (N = 5 per group) on either an AIN-93 diet or high fat diet (45% kcals from fat). Values are expressed as means±SE, different letter superscripts indicate statistical significance (p<0.05).

Significant differences in both EE and RER between offspring of obese and lean dams which were further exacerbated when challenged with a high fat diet as shown by the EC50 values in **Figure 1A and 1B**. As expected, increases in EE were observed in both groups in response to the HF diet. However, EE was reduced in offspring from obese dams on both control (p<0.001, 52.36±0.07 vs. 50.98±0.05 kcals/day), and high fat diet (p<0.001, 61.28±0.08 vs. 59.20±0.12 kcals/day) as compared to offspring from lean dams. 24-h averaged values showed increased EE in both offspring groups in response to HF diet (**Figure 1C**), but was not sensitive enough to detect the differences due to maternal obesity as shown by PRCF in **Figure 1A**. PRCF analysis also showed differences in RER between offspring groups and by type of diet. As expected, the HF diet caused RER values to decline in both the offspring of lean and obese rat dams. On the AIN-93G diet, RER values of offspring were greater in obese offspring as compared to lean offspring (0.96±0.0004 vs. 0.98±0.0002, p<0.001). When challenged with a high fat diet the decline in RER was blunted in the offspring from obese dams with an RER value of 0.91±0.0002 vs. 0.94±0.0002 in offspring from lean dams (p<0.001). No differences in average 24 h RER values (**Figure 1D** **)** were observed in offspring of obese dams when fed a control or HFD. Total activity measures of rats during indirect calorimetry revealed no effect of maternal obesity in the offspring; however, both offspring groups showed significantly reduced total activity when fed a HF diet (**Figure 1E**). Food intake was also measured every other day and there were no differences between offspring due to maternal obesity on either diet (**Figure 1F**).

### Hepatic Sirtuin (SIRT) mRNA Expression is Influenced by Maternal Obesity

**Figure 2:**
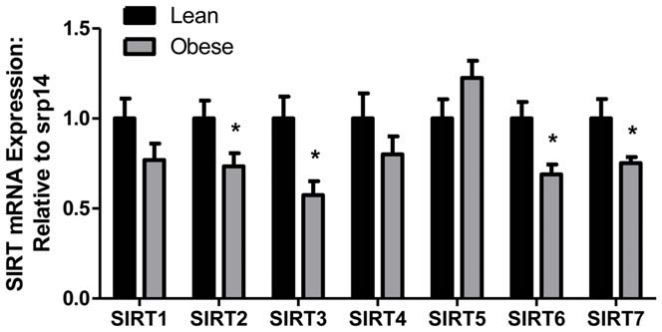
Hepatic mRNA expression of sirtuin 1-7 from offspring of lean and obese dams at PND 21. Gene expression was assessed via real-time RT-PCR (N = 7 per group). Values are expressed as means±SE. Statistical differences were determined using a Student's t-test. * indicates p<0.05.

To investigate if changes in EE and RER in offspring of obese dams were related to sirtuin expression, we examined hepatic mRNA expression of the sirtuin family (SIRT1, 2, 3, 4, 5, 6, and 7). At PND21 offspring of obese dams showed reduced mRNA expression of SIRT2 (27%, p = 0.052), SIRT3 (43%, p = 0.012), SIRT6 (31%, p = 0.013), and SIRT7 (25%, p = 0.049) as compared to lean dam offspring (**Figure 2**). Differences in mRNA levels of SIRT1, SIRT4, and SIRT5 between lean and obese groups did not reach statistical significance.

### Mitochondrial Protein Content of SIRT3 and Electron Transport Chain (ETC) Complexes

**Figure 3:**
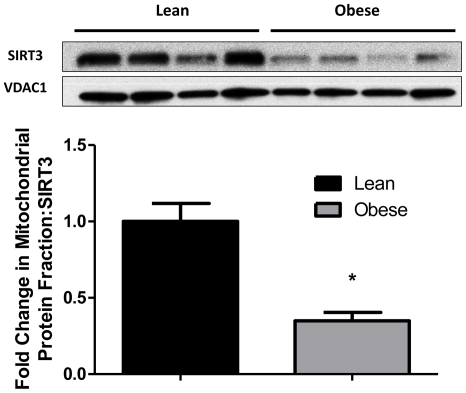
Hepatic SIRT3 mitochondrial protein content of lean and obese dam offspring. Representative blot and densitometric quantitation of SIRT3 protein content in the mitochondrial fraction from livers of offspring of lean and obese dams at PND21 by Western blotting (N = 4 pools representing a total of 8 animals/group). Values are expressed as means±SE. Statistical differences were determined using a Student's t-test. * indicates p<0.05.

**Figure 4:**
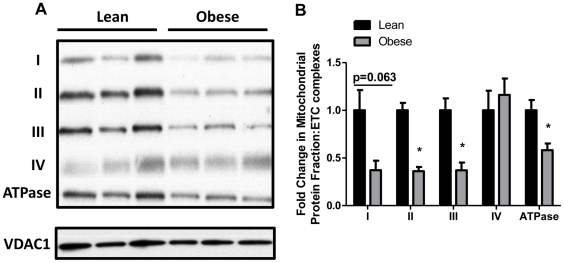
Electron transport chain complexes from mitochondrial fractions of livers from offspring of lean and obese dams at PND 21. (A) Representative blots and (B) densitometric quantitation (N = 3–4 pools representing a total of 6–8 animals/group). Values are expressed as means±SE. Statistical differences were determined using a Student's t-test. * indicates p<0.05.

Since SIRT3 is primarily localized in the mitochondria and is critical for fatty acid oxidation, we investigated the levels of SIRT3 protein in mitochondrial extracts. Consistent with gene expression data, SIRT3 protein levels were also markedly reduced (∼3-fold) in offspring of obese dams (p = 0.002) as shown in **Figure 3**. Several components of the ETC are highly regulated by nutritional status (e.g. fasting) through acetylation of key residues which are downstream targets of SIRT3. Representative blots of the five electron transport chain complexes are shown in **Figure 4A**. Apoprotein levels of complexes II (p = 0.001), III (p = 0.012), and ATPase (p = 0.031) were reduced by 64%, 63%, and 42% respectively in the offspring of obese dams as compared to lean dam offspring (**Figure 4B**). Decreases in levels of complex I nearly reached statistical significance (p = 0.063) in offspring of obese dams. To indirectly estimate mitochondrial content we assessed mRNA levels of mitochondrial transcription factor A (mtTFAM). No differences were observed in TFAM mRNA between offspring of lean and obese groups (data not shown).

### Fasting-Induced SIRT3 and PGC1α mRNA Expression and Protein Is Blunted in Offspring of Obese Dams

**Figure 5:**
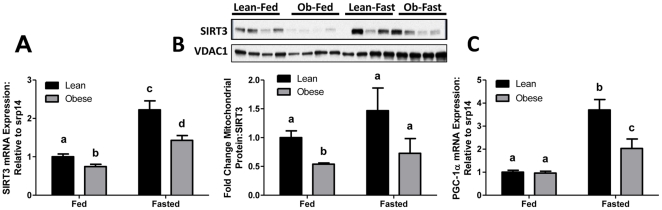
Fasting-induced changes in SIRT3 and PGC-1α mRNA and protein expression of lean and obese dam offspring. (**A**) SIRT3 mRNA expression, (**B**) SIRT3 mitochondrial protein content, and (**C**) PGC-1α mRNA expression in liver from fed and fasted offspring of lean and obese dams at PND 21 (N = 8–15 per group). Representative blot is also shown in FIG 5B for SIRT3 protein content (N = 4 per group). Values are expressed as means±SE, different letter superscripts indicate statistical significance (p<0.05).

SIRT3 and peroxisome proliferator activated receptor-γ coactivator (PGC)-1α are critical regulators of mitochondrial fatty acid oxidation that are sensitive to nutritional changes and are particularly induced by fasting. Since fasting robustly activates pathways involved in fatty acid mobilization, we utilized this challenge to discern differences between offspring of lean and obese dams that may be evident during fasting. In the fed state, we observed 25% lower SIRT3 mRNA expression in the offspring from obese dams (p = 0.011) as compared to offspring from lean dams (**Figure 5A**). As expected, fasting led to increased expression of SIRT3 mRNA in offspring of lean dams (122%, p<0.001). However, while still greater than fed controls (p<0.005), the fasting induction of SIRT3 mRNA in offspring of obese dams was blunted as compared to the induction that occurred in offspring of lean dams (p<0.001) indicating a deficit in the key regulators of fatty acid mobilization (**Figure 5A**). Protein content of SIRT3 in the mitochondrial fraction under fed conditions mirrored mRNA expression in **Figure 5B**. Trends towards a fasting-induced increase in SIRT3 protein as compared to the fed state, in offspring of lean dams, as well as towards reduced SIRT3 protein in the offspring of obese dams were preserved, consistent with gene expression data (**Figure 5B**). However, these differences did not attain statistical significance. While PGC1α mRNA levels did not differ between offspring of lean and obese dams under fed conditions, fasting elevated PGC1α mRNA expression (3.7-fold, p<0.001) in the offspring of lean dams (**Figure 5C**). However, similar to SIRT3 mRNA expression, there was a blunted fasting-associated increase of PGC1α mRNA expression (2-fold, p<0.001) in the offspring of obese dams. These findings suggest that basic transcriptional responses coordinating fasting-associated fatty acid oxidation are impaired by exposure to maternal obesity, consistent with the aforementioned phenotypic (fatty liver) and physiological changes (EE and RER).

### Long Chain Acyl-CoA Dehydrogenase (LCAD) Mitochondrial Protein Content

**Figure 6:**
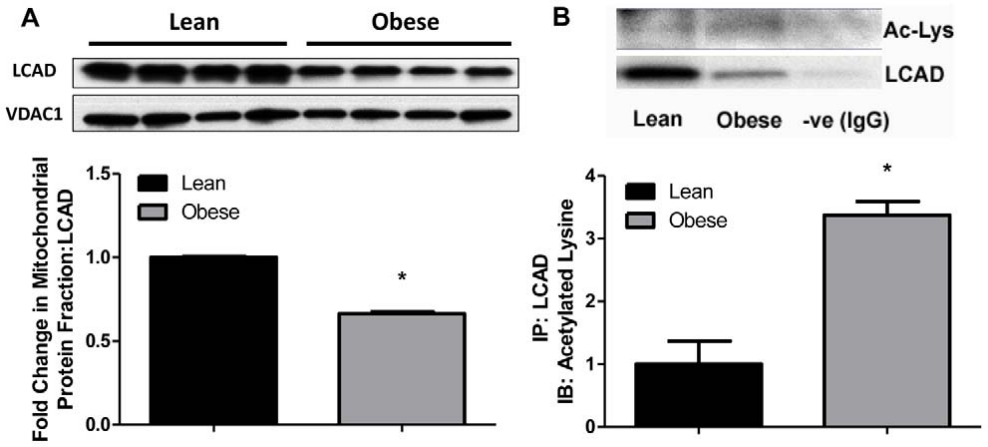
Hepatic mitochondrial protein content and acetylation of LCAD of lean and obese dam offspring. (**A**) Representative blot and densitometric quantitation of LCAD in the mitochondrial fraction from livers of offspring from lean and obese dams at PND21 by Western blotting (N = 4 pools representing a total of 8 animals/group). (**B**) Representative blot and densitometric quantitation of acetylated LCAD in the mitochondrial fraction from fasted livers of offspring at PND21 (N = 3 per group). Immunoprecipitation of LCAD was performed in total liver lysates and immunoblotting was performed using anti-acetylated lysine antibody. Values are expressed as means±SE. Statistical differences were determined using a Student's t-test. * indicates p<0.05.

LCAD is a key enzyme involved in β-oxidation that is highly regulated by SIRT3. Mitochondrial protein content of LCAD was reduced in the offspring of obese dams (p<0.001) as shown in **Figure 6A**. Further, we found that maternal obesity led to hyperacetylation of LCAD indicating reduced deacetylase activity of SIRT3 (**Figure 6B**).

## Discussion

The precise mechanisms underlying increased susceptibility to excessive weight gain and adiposity of offspring from obese women remain unclear. In the present work, we investigated alterations in hepatic and whole body energy metabolism in the offspring from lean and obese rat dams at weaning, prior to differences in body weight or adiposity. Our studies reveal several salient findings. First, maternal obesity decreased offspring energy expenditure and favored decreased efficiency to utilize fatty acids as fuel substrate when offspring were fed either a control (AIN-93G) or high fat diet (45% kcal from fat) based on heat and RER values. Second, our results suggest hepatic mitochondrial dysfunction in both fed and fasted states. This was associated with impaired SIRT3/PGC1a induction in fasting levels and dysregulation of fatty acid oxidation and electron transport chain complexes. Together, these findings suggest impaired nutrient sensing and fuel switching in offspring from obese dams.

Indirect calorimetric assessments revealed a modest decrease in energy expenditure in the offspring of obese dams fed either a control or HFD at weaning (**Figure 1A**). It has been previously reported by others that minimal differences in energy balance can lead to obesity over time [27]–[29]. The current studies focused solely on young offspring to ascertain differences in metabolism prior to divergence in body weight. We did not expect marked differences in EE between offspring, but sought to determine if there were subtle, but detectable differences in EE as early as PND21. It is important to note there were no differences in body weight or body composition at PND21 between lean and obese dam offspring [9], [10]. However, the decrease in EE seen in obese dam offspring (**Figure 1A**) was accompanied by a trend towards increased body weight gain (40.2±3.3 g vs. 44.2±1.7 g, p = 0.297, N = 5 per group) on HFD (over 4 d) as compared to lean dam offspring. Moreover, obvious divergence of body weight in the offspring does not appear until PND60 [9]. Hence, it is likely that the offspring have a significant energy imbalance during adulthood. Together, these current and previous findings suggest offspring from obese dams are less able to adapt their energy expenditure in the face of increased caloric intake and are thus susceptible to obesity. However, we plan to measure EE in adult offspring of lean and obese dams during divergent weight gain (PND60) to confirm if changes in EE persist and directly contribute to the development of obesity. Our data is consistent with a study by Rising and Lifshitz (2008) that showed decreased EE and increased adiposity in infants of obese mothers as compared to infants born to lean mothers [30].

A hallmark of greater reliance on fatty acids as an energy source (either during fasting or consumption of HF diets) is the lowering of RER values [31]–[34]. Offspring from obese dams uniformly showed small but consistently higher RER values on either control or HF diets. Both greater *de novo* lipogenesis and impaired fatty acid utilization could presumably account for higher RER values in offspring of obese dams. In a recent report, we demonstrated that obese dam offspring display hepatic steatosis and a lipogenic transcriptomic signature associated with greater sterol regulatory binding protein (SREBP)-1c and lower peroxisome proliferator activated receptor (PPAR)-α/5′-AMP-activated protein kinase (AMPK) signaling at weaning. The present data from indirect calorimetry are consistent with our previous report. The differences in RER values between lean and obese dam offspring were greater when challenged with HF diets, suggesting impaired metabolic flexibility (i.e. to adapt substrate utilization with substrate availability) [35] (**Figure 1B**). In the current report, we focused on examining mechanisms regulating fatty acid oxidation that may explain this inflexibility. A recent study conducted in obese adolescents (11–18 yr old) with non-alcoholic fatty liver disease (NAFLD) reported that hepatic fat accumulation led to decreased reliance on fatty acid oxidation in the fasted state. This was accompanied by an inability to suppress fatty acid oxidation (FAO) during an oral glucose tolerance test as determined by RER values. This impaired capacity to switch substrate utilization to FAO during fasting and back to carbohydrate oxidation when glucose challenged indicates metabolic inflexibility [36]. Most importantly, impaired FAO was determined by hepatic fat content and not abdominal adiposity. Hence, it appears that there is an intricate relationship between hepatic steatosis and fatty acid oxidation. Consistent with these findings, offspring from obese dams develop increased liver weight and hepatic fat accumulation without differences in body weight or adiposity [10]. Therefore, it is plausible that exposure to maternal obesity alters metabolic sensors leading to an impaired ability to oxidize fat.

Mitochondria are typically the primary site for FAO, and since mitochondria are maternally inherited, several models of gestational programming have focused on changes in this organelle [13], [14], [37]–[41]. Since our model exclusively examines the contribution of maternal obesity, mitochondrial changes may be an important conduit on how maternal obesity mediates programming of offspring metabolism. Mitochondrial dysfunction is highly associated with reduced FAO [11], [12], [14], [15]. While we found no differences in mRNA expression of mitochondrial transcription factor A (mtTFAM) between offspring of lean and obese dams, suggesting that mitochondrial numbers may not be affected, our studies identified several indications of mitochondrial dysfunction, including lower abundance of oxidative phosphorylation (OXPHOS) complexes (**Figure 4**). In addition to lower amounts of OXPHOS proteins, the function of the electron transport chain complexes and other mitochondrial proteins are highly regulated post-translationally via lysine acetylation [42]. Recent studies have shown SIRT3, a member of the class III NAD+ dependent deacetylase family, to be located in the mitochondria and known to critically regulate OXPHOS in the liver [43], [44].

Sirtuins act as energy sensors and regulate metabolic processes via their deacetylation activity. The sirtuin family consists of seven isoforms (SIRT1-7) that regulate distinct metabolic pathways in various cellular locations [45]. SIRT1, 6, and 7 are located in the nucleus, SIRT2 in the cytosol, and SIRT3, 4, and 5 in the mitochondria [45]. Our data suggest that maternal obesity affects the levels of several SIRT isoforms (SIRT2, 3, 6, and 7) in the offspring liver (**Figure 2**), suggesting that the SIRT family may play a role in fetal metabolic programming. We chose to examine SIRT3 due to its mitochondrial location and lack of change in mRNA expression in the other mitochondrial located isoforms (SIRT 4, 5). Further, Lombard et al. found that SIRT4- and SIRT5-deficient mice did not increase global lysine acetylation in contrast to SIRT3-deficient mice [46]. Elegant studies in early mouse embryos and blastocyst also reveal that SIRT3 is maternally inherited and critical for protection from reactive oxygen species [47]. Moreover, SIRT3 has been associated with increasing energy utilization in liver [48], skeletal muscle [49], and brown fat [50] suggesting a role in increased whole body energy expenditure and is highly responsive to dietary challenges such as high fat diet or fasting. Studies also show that SIRT3 mRNA expression and protein content in the liver are decreased in response to nutrient excess [44] and increased in response to fasting [48]. Hence, given the critical roles for SIRT3 in multiple aspects of fat and energy expenditure, programming of SIRT3 may have important consequences for offspring metabolism. Utilizing SIRT3-knockout (KO) mice, Hirschey et al. performed a meticulous study demonstrating the role of SIRT3 in regulating mitochondrial fatty acid oxidation (FAO) [48]. Increased SIRT3 expression, in response to fasting, induced LCAD via deacetylation leading to increased FAO in the liver, heart, and brown fat. Moreover, overexpression of SIRT3 rescued hepatic FAO in the SIRT3 KO mice. Our results from offspring of obese dams are analogous to the phenotypic changes observed in the SIRT3 KO mice (elevated RER, reduced SIRT3 mRNA and protein, and hyperacetylation of LCAD) would strongly suggest that hepatic FAO may be reduced in the offspring of obese dams. Further, a recent study by Kendrick et al. showed that fatty liver is associated with decreased SIRT3 activity, hyperacetylation of key mitochondrial proteins, and impairment of the ETC [44]. These data are again consistent with previously reported hepatic steatosis and lipid accumulation in offspring of obese dams at weaning [10].

Deficits in FAO in offspring of obese dams are certainly not limited to lower SIRT3 and mitochondrial OXPHOS. We previously reported that carnitine palmitoyl-CoA transferase (CPT)-1, the rate-limiting enzyme for fatty acid entry into the mitochondria, is reduced in the offspring of obese dams [10]. This was associated with a coordinated down-regulation of PPAR-α regulated genes and reduced phosphorylation of AMPK^Thr172^ in the offspring of obese dams. Phosphorylation of AMPK induces activation of catabolic processes such as glucose uptake and fatty acid oxidation [10] and has been shown to be affected in other models of maternal overnutrition [51]–[53]. Moreover, SIRT3 appears to regulate AMPK activation as shown in skeletal muscle [49] and human hepatic cells [54]. Further, Pillai et al. have recently reported that the regulatory mechanism is via SIRT3 deacetylation and activation of LKB1, an upstream kinase known to activate AMPK in mice hearts [55]. It is likely that a reduction in hepatic fatty acid oxidation not only further reinforces mitochondrial dysfunction, but may also be contributing to the development of hepatic steatosis observed in the offspring of obese dams at weaning [10].

Adaptation to fasting requires activation of numerous pathways that coordinate the mobilization of fatty acids. Upregulation of PPAR-α is one of the primary drivers in the liver. It has been previously reported that mice deficient in PPAR-α develop dramatic hepatic steatosis upon fasting [56]–[58]. Increases in pyruvate and nicotinamide adenine dinucleotide (NAD)+ levels during fasting result in greater enzymatic activity and protein content of SIRT1 in the nucleus [59], [60]. Among its many actions, SIRT1 activates PGC-1α via deacetylation leading to transcriptional activation of a complement of genes associated with mitochondrial biogenesis [61], [62], OXPHOS and fatty acid oxidation [63]–[65]. Interestingly, it appears that PPAR-α acts upstream of SIRT1, although the precise mechanisms remain unknown [60]. SIRT1 also antagonizes lipogenic gene expression, mainly via SREBP-1. Andenovirus-mediated hepatic overexpression of SIRT1 in mice during fasting significantly downregulated SREBP-1c, fatty acid synthase (FASN), and elongation of very long chain fatty acids (ELOVL)-6 [66]. Offspring of obese dams have greater lipogenic gene expression via SREBP-1c [10] and while SIRT1 mRNA was not significantly altered in offspring of obese dams, a more detailed analysis of SIRT1-mediated regulation of lipogenesis is certainly warranted. Consistent with our earlier findings on PPAR-α [10], the current data show that maternal obesity led to blunted fasting-mediated induction in both SIRT3 and PGC-1α mRNA expression in the offspring. While the precise crosstalk between SIRT3 and PGC-1α is still being actively investigated, SIRT3 promotes the expression of PGC-1α in brown fat [50] and SIRT3 deficient mice express decreased mRNA levels of PGC-1α in skeletal muscle [49]. Furthermore, a recent study also showed that PGC-1α positively regulated SIRT3 gene expression in myocytes and hepatocytes, via direct recruitment to the SIRT3 promoter via an estrogen receptor-related-α binding site [67].

In conclusion, we have shown that maternal obesity contributes to early perturbations in whole body and liver energy metabolism in the offspring at weaning. Decreased expression of SIRT3 and other key mitochondrial proteins involved in fatty acid oxidation and OXPHOS suggest mitochondrial dysfunction may precede more detrimental obesity associated co-morbidities such as insulin resistance and NAFLD.
